# Supplementary material for: Nutritional Nesting (Nestrition): Shaping the Home Food Environment in the First Pregnancy
Source: Nutrients. 2024 Sep 30;16(19):3335. doi: 10.3390/nu16193335 (PMC11478405; doi:10.3390/nu16193335)
Supplement: Supplementary file 1 [file nutrients-16-03335-s001.zip › Supplementary S1. Interview guide.pdf]

Interview guide.

Subject:			part 1- interview guide:	Time:
Introduction:			**Nice to meet you. I would like to thank you for participating in this study. Would you like me to tell you a bit more about the research?**	5
***First pregnancy**	1	*a*	**Can you please tell me a bit about yourself and how your pregnancy is going so far?**<br>(How are you feeling about being pregnant? Have you had any changes in your appetite or sleep habits? (If yes) What?)	
*** As a parent**			**Can you please tell me a bit about your childbirth and how your parenting life is going so far?**<br>**(How are you feeling about being a parent? Have you had any changes in your appetite or sleep habits? (If yes) What?)**	"
**PHFE**-<br>Knowledge	2	*a*	**The food we have at home is all part of our 'home food environment'.**<br>**What do you think is meant by a 'healthy home food environment'?**	30
Perceive own PHFE		*b*	**How healthy do you think <u>your</u> 'home food environment' is now?**	
Teachable moment		*c*	**How <u>important</u> is it that your 'home food environment' is healthy now (in pregnancy)? Why? Are you able to share some examples of things that are important to you?**	
			**How important is it that your 'home food environment' is healthy now (in early parenthood)? Why? Are you able to share some examples of things that are important to you?**	
		c-	**Can you compare it to the time when you were pregnant?**	
Barriers		*d*	**How easy or difficult is it for you to maintain a healthy PHFE? Can you say a bit more about any difficulties?**	
Food dessert			**Are there any particular obstacles to GETTING the food you want or need? What could help you overcome these obstacles?**	
Food preparations			**Are there any obstacles that keep you from PREPARING the food you would like to eat? Do you have any barriers that prevent you from cooking? What could help you overcome them?**	
Levers		*e*	**What <u>motivates</u> you to keep a healthy 'home food environment'?**	
1		*f*	**Where does your <u>partner stand regarding</u> 'home food environment'? (before pregnancy**/being a parent, **now, and in the coming future)**	
Intention to change		*g*	**Do you intend to make any changes yourself during the pregnancy? Why?**	
Comparison		*h*	**Is your 'home food environment' different now to before you learnt you were pregnant**/ had your baby? **Can you give me some examples?**	
**Nestrition**	3	*a*	**How important do you think preparing a healthy 'home food environment' is for first time pregnant couples? Why?**	10
To probe from the questionnaire		*b*	**Do you think it would be a good idea to have classes for couples during pregnancy/**first time parents **about a healthy 'home food environment'?**	
		c	**What do you think would be a good format for such a program?**<br>*(partners-in what stage of preg-group or private-no& length of meetings-food preparation- home work- Emphasis*)	
**Consumption**	4		Overall, how healthy do you think is what you eat at the moment?	15
			To go through the food diary.	
**PHFE**	5		Questions to probe from the questionnaire- SEE BELOW*	
**Comments**	6		**Is there anything else that you can add to help me? Do you have any other questions or comments?**	
Conclusion:			**I wish to thank you for your time. If following the interview you feel you wish to further share any information with me, or to chat, please do not hesitate to contact me. In a few months, I will contact you again, to ask you to join in one of our focus groups, when your baby is around 3 to 4 months old. I would like to hear how you are doing and whether your views have changed. May I wish you all the best in your special coming future.**	
*			**I wish to thank you for your time. If following the interview, you feel you wish to further share any information with me, or to chat, please do not hesitate to contact me. Please accept the 40£ voucher as a token of appreciation for your participation.**<br>**May I wish you many happy, wonder-filled times ahead, you deserve every bit of happiness this baby is going to bring you!**	

*** Part 2- questions To probe from the questionnaire:**

Room scape : = the room or bounded setting where food is prepared and consumed

a. **Kitchen appliances- use and accessibility.**

b. **What foods do you stockpile at home, why?**

c. **Do you have enough space in the kitchen to prepare healthy food?**

PHOTOS: walls+ all the accessible kitchen-scape

Table scape : = the furniture or surface from which food is consumed

a. **Do you have enough space at home to eat family meals together (dining table)?**

b. **What is currently on your dining table now?**

PHOTOTS- tables (in kitchen& dining room)- size, type, clutter, table access, accommodations, amenities

Food scape: = the edible item that are consumed.

a. **Usually, who decides what food to cook for the family?**
- Just you
- Only your partner
- You and your partner decide together
- Other (e.g., grandmother, other family member)

b. **Could you please describe me how you cooked the last meal cooked at home?** *(Margaret Raber's…)*

c. **On average, how often do you eat fast food from places such as McDonald's, KFC and Subway**? This includes both eating in and taking food away from fast food places

d. **On average, how often do you eat other convenience foods for your main meal?** This includes food that requires no preparation such as ready-made pizza, microwaveable meals, fish fingers, chicken nuggets and takeaway food such as fish and chips, Chinese, and Indian.

e. **Do you have any kinds of snack foods that you make at home?**

f. **If yes, please name the types of snack foods that you make at home:**

g. **Which of the following beans (canned, dried or frozen) are in your home right now:**
- Black beans
- Black-eyed peas
- Chickpeas or garbanzo beans
- Lima beans
- Pinto beans
- Northern beans
- Kidney beans
- Split peas
- Baked beans
- Refried beans
- Other, please specify

h. **Which of the following grain products do you have in your home now?**
- Brown rice (whole grain).
- Any Cereals grains other than wheat (e.g. barley, buckwheat, quinoa, millet).
- Oats
- Legumes (e.g. lentils, chick peas, soy, dried beans etc.)
- Seeds (e.g. Sunflower, flax, sesame, pumpkin, seeds)
- Flour.
  -If you have flour, what type do you have:
- Whole Cereals grains (e.g. whole wheat, barley, buckwheat, quinoa, millet).

i. **Which of the following oils do you have in your home now?**
- Olive oil
- Liquid oil. Type:______
- Coconut oil
- Butter
- Margarine
- Other:

j. **Usually, who does the food shopping for the family?**
- Just you
- Only your partner
- You and your partner decide together
- Other (e.g., grandmother, other family member)

k. **Shopping habits:**
- Where do you choose to shop for food? Why? Where else?
- On average, what is your monthly budget for groceries?
- How often do you buy groceries? (how often do you shop for food)
- How do you travel to the supermarket? How long to get there?

l. **Do you have baking soda (also known as sodium bicarbonate) at home, and what do you use it for?**

PHOTOS- foods displayed out in the open, without opening any opaque cupboard doors + in the fridge

General:

a. Do you currently attend or intend to go to antenatal classes?

b. After you have the baby, how easy or difficult do you think will it be for you to prepare healthy nutritious food for him/her?

c. Is your general Health different then before the pregnancy?

d. Which of these descriptions fit you best (pre pregnancy): underweight—healthy weight—a bit overweight—very overweight--obese

e. Have you had your weight checked recently? lb Date : Height: (feet and inches)
