# Supplementary material for: Nutritional Nesting (Nestrition): Shaping the Home Food Environment in the First Pregnancy
Source: Nutrients. 2024 Sep 30;16(19):3335. doi: 10.3390/nu16193335 (PMC11478405; doi:10.3390/nu16193335)
Supplement: Supplementary file 1 [file nutrients-16-03335-s001.zip › Supplementary S2. Questionnare_Women.pdf]

UNIVERSITY OF LEEDS

Bar-Ilan University

Home Food Environment of first-time pregnancy - PRIME questionnaire:

Thank you for showing interest in this study. You are being invited to participate in a research study called *PRIME*. The aim of this study is to explore the Home Food Environment of First-Time-Pregnant couples and it will take you about 30 minutes to complete. The study is being carried out by Chagit Peles with a group of other researchers from the University of Leeds and Bar-Ilan University.

Your participation in this study is highly appreciated and entirely voluntary. You can withdraw from the study at any point and if you do all your information will be destroyed.
You do not have to answer any questions you do not want to.

Please choose one answer for each question, unless indicated otherwise:

As a participant in this survey you can participate in prize draw valued at £100.
Please indicate if you wish to participate in the prize draw:

- o **I wish** to participate in the prize draw.
- o **I do not wish** to participate in the prize draw.

1. **Overall, how healthy do you think your lifestyle is at the moment?**

☹ *not at all* — 😐 — *Very healthy*☺

1	2	3	4	5	6	7	8	9	10

2. **What kind of cooking do you do at the moment?** *(Please tick as many boxes as appropriate)*
   - ☐ Cook convenience foods and ready-meals.
   - ☐ Put together ready-made ingredients to make a complete meal (e.g. Use ready-made sauces).
   - ☐ Prepare dishes from basic ingredients.
   - ☐ Other, please specify: ___________________
   - ☐ Don't cook at all.
   - ☐ My partner does the cooking usually.

3. **In a normal week, how often do you prepare and cook a main meal from basic ingredients**, For example: making Shepherd's Pie starting with raw mince and potatoes?
   - ☐ Daily.
   - ☐ 4-6 times a week.
   - ☐ 2-3 times a week.
   - ☐ Once a week.
   - ☐ Less than once a week.
   - ☐ Never.

4. **How confident do you feel about being able to cook from basic ingredients?**

Extremely Confident	1	2	3	4	5	6	7	*Not at all Confident*

5. **How confident do you feel about following a simple recipe?**

Extremely Confident	1	2	3	4	5	6	7	*Not at all Confident*

6. **How confident do you feel about tasting foods that you have not eaten before?**

Extremely Confident	1	2	3	4	5	6	7	*Not at all Confident*

7. **How confident do you feel about preparing and cooking new foods and recipes?**

Extremely Confident	1	2	3	4	5	6	7	*Not at all Confident*

8. When you are cooking, how often do you do the following:

a) I measure the amount of oil or fat I use:

Almost never	Only occasionally	Often	Always

b) I use low fat cooking methods like – baking, boiling, steaming, grilling:

Almost never	Only occasionally	Often	Always

c) I use olive oil when cooking:

Almost never	Only occasionally	Often	Always

d) I use herbs/spices/lemon/onion/garlic for preparing meal:

Almost never	Only occasionally	Often	Always

e) I fry food (For example, chips, deep fried chicken):

Almost Never	Only occasionally	Often	Always

f) I add sugar or sweeteners while cooking:

Almost never	Only occasionally	Often	Always

g) I use processed meats when cooking [like bacon/sausage/hotdogs]:
(For example, use bacon to flavour a soup).

Almost never	Only occasionally	Often	Always

h) I add fresh or frozen fruit or vegetables (not canned) to the main meal, not just veg side dishes:
(For example – Add fresh carrots or tomatoes to rice).

Almost never	Only occasionally	Often	Always

i) I add a small amount or no salt when cooking:

Almost never	Only occasionally	Often	Always

9. In anticipation of the birth of your baby, how interested would you be in attending classes/workshops preparing you for the coming future after you become a parent. That includes five aspects of healthy lifestyle for a new family (for both parents and child):

	Not interested				*Very much interested*
• **Parenting**	1	2	3	4	5
• **Nutrition**	1	2	3	4	5
• **Physical activity**	1	2	3	4	5
• **Sleep**	1	2	3	4	5
• **Coping with stress & Emotional wellbeing**	1	2	3	4	5
• **Other:**	1	2	3	4	5

10. If you were offered free classes on those topics:

a) **At what point in time would you be interested?**

Not interested	1st trimester	2nd trimester	3rd trimester	Once I am a parent

b) How many meetings do you think people like you would like to have overall?

Not interested	1 class	2 classes	3 classes	4 classes or more

c) How much do you think is reasonable to pay for each class?

Nothing	up to £ 5	£ 5 to £ 10	More than £ 10	'Pay what you can'

d) Do you think your partner would be interested in participating?

Not interested				*Very much interested*
1	2	3	4	5

11. *FRESH FRUIT:*

Which of the following fruits are in your home now? *(Please tick as many boxes as appropriate)*

- ☐ Apples
- ☐ Bananas
- ☐ Blueberries
- ☐ Cherries
- ☐ Grapefruit
- ☐ Grapes
- ☐ Kiwi
- ☐ Mangoes
- ☐ Melon / Watermelon
- ☐ Peaches/Nectarines
- ☐ Oranges/tangerines/satsumas/mandarins
- ☐ Pears
- ☐ Pineapple
- ☐ Plums
- ☐ Strawberries
- ☐ Other fresh fruit (please specify):__________

12. Would you say that the amount of fruit you currently have in your home is more than usual, less than usual, or about the same?

Less than usual	The same	More than usual

13. Without opening any opaque cupboard doors, is there any kind of fruit in your home now, displayed out in the open?

(If some fruit is behind a door, but it is a glass door and the fruit can be seen, tick YES)

Yes	No

14. *FRESH VEGETABLES*:

Which of the following vegetables are in your home now? *(Please tick as many boxes as appropriate)*

- ☐ Asparagus
- ☐ Bell Peppers (green, orange, red, yellow)
- ☐ Broccoli
- ☐ Brussels sprouts
- ☐ Butternut squash
- ☐ Carrots
- ☐ Cabbage
- ☐ Celery
- ☐ Corn on the cob
- ☐ Cauliflower
- ☐ Cucumber
- ☐ Green beans/String beans/Runner beans
- ☐ Green, leafy veg (e.g. kale, collard greens, watercress, Swiss chard)
- ☐ Lettuce
- ☐ Mushrooms
- ☐ Onions
- ☐ Potatoes
- ☐ Pumpkin
- ☐ Spinach
- ☐ Sweet potatoes or yams
- ☐ Tomatoes – standard size
- ☐ Tomatoes – cherry or grape
- ☐ Courgettes
- ☐ Swede
- ☐ Other fresh vegetables (*please specify*):_________

15. Would you say that the amount of vegetables you currently have in your home is more than usual, less than usual, or about the same?

Less than usual	The same	More than usual

16. Do you have any ready-to-eat fresh vegetables on a shelf in the fridge or on the kitchen counter now? (These include baby carrots, cherry tomatoes, or salad vegetables that you have sliced to make them ready to eat).

Yes	No

17. Which of the following kind of snack foods do you have in your home now?

(Please tick as many boxes as appropriate)

- ☐ **Cheese puffs or Wotsits.**
- ☐ **Crisps (potato, corn, tortilla, pita, or vegetable chips).**
  - How many different flavours of crisps are in your home (such as plain, barbeque, sour cream and onion, nacho cheese, Doritos, lime)? ____

    (For example, if you have one bag of Doritos, one bag of plain potato chips, and one bag of sour cream and onion potato chips, you would record "3".)
- ☐ **Pretzels.**
- ☐ **Popcorn:**

  If you have popcorn, is it *[mark all that apply]*:
  - ☐ Popped
  - ☐ Unpopped
- ☐ **Rice cakes.**

☐ **Crackers:**
If you have crackers, what type are they? *[Mark all that apply]*
☐ Wholemeal
☐ Low fat
☐ Regular
☐ Home-made crackers
☐ **Biscuits/Cookies:**
- How many different types of biscuits are in your home (such as chocolate chip, oatmeal raisin, Oreo, biscuits, digestive biscuit, etc.)?____
(For example, if you have chocolate chip cookies, Oreos, and animal biscuits, you would record "3".)
☐ **Cakes, pies, cupcakes, brownies, rice Krispy treats.**
☐ **Muffins/sweet breads** (such as banana)**.**
☐ **Pastries/cinnamon rolls/doughnuts:**
- How many different types of these foods are in your home (such as coffee cake, cheese cake, cupcakes, banana muffins, Swiss cake rolls, etc.)? ____
(For example, if you have banana muffins, Twinkies, and toaster strudel cupcakes, Swiss roll, you would record "3".)
☐ **Candy / Sweets:**
- If you have candy / sweets, what type do you have? *[Mark all that apply]*
☐ Chocolates
☐ Hard candy such as boiled sweets
☐ Chewy sweets
☐ Other
- How many different types of candy / sweets are in your home? ____
(For example, if you have chocolate bars, Milky Way, toffees, boiled sweets, liquorice allsorts, you would record "5".)
☐ **Puddings, gelatin desserts.**
☐ **Gummy-fruit candies, fruit snacks, fruit roll-ups.**
☐ **Granola/cereal bars.**
☐ **Ice-cream (regular or low-fat):**
- How many different flavours of ice cream do you have in your home (such as chocolate chip, vanilla, strawberry)? ____
☐ **Frozen yogurt:**
- How many different flavours of frozen yogurt do you have in your home (such as chocolate chip, vanilla, strawberry)? ____
☐ **Ice lollies:**
If you have Ice lollies, are they regular, 100% juice or both? *[Mark all that apply]*
☐ Regular
☐ 100% juice

18. Would you say that the amount of snacks you currently have in your home is more than usual, less than usual, or about the same?

Less than usual	The same	More than usual

19. Which of the following kinds of beverages do you have in your home now?
(Please tick as many boxes as appropriate)
☐ **Fizzy drinks** [including Coke, Pepsi, Sprite as well as flavoured waters or Schweppes with sugar]
- How many different types do you have in your home (such as cola, sprite, ginger ale, lemonade)? ____
☐ **Diet fizzy drinks** [including Coke Zero, Diet Pepsi, as well as 0 calorie flavoured waters or seltzers]
- How many different types do you have in your home (such as cola, sprite, ginger ale, lemonade)? ____
☐ **Sweetened drinks** [such as, Sunny Delight, , sweet tea, Snapple]
- How many different types of sweetened drinks do you have in your home? ____
☐ **Juice drink syrup** [such as Ribena, fruit cordials]**.**
☐ **100% fruit juice or fruit and vegetable combination.**

20. Would you say that the amount of beverages you currently have in your home is more than usual, less than usual, or about the same?

Less than usual	The same	More than usual

21. How often do you shop for food? ______

22. How many days has it been since you last shopped for food? ____ **Days**.

(If you do online shopping, which is then delivered, answer how many days it has been since food was last delivered to your house, as we want to know how many days it has been since food shopping came into the house.)

23. Was the last shop small or big?

Small	Medium	Big

.

24. During a typical WEEKDAY,
how often is the TV (or any screen) on during your meals, even if you are not watching it?

• Breakfast	Never	Rarely	Sometimes	Often	Always
• Snack	Never	Rarely	Sometimes	Often	Always
• Lunch	Never	Rarely	Sometimes	Often	Always
• Dinner / Supper	Never	Rarely	Sometimes	Often	Always

25. During a typical WEEKEND DAY,
how often is the TV (or any screen) on during your meals, even if you are not watching it?

• Breakfast	Never	Rarely	Sometimes	Often	Always
• Snack	Never	Rarely	Sometimes	Often	Always
• Lunch	Never	Rarely	Sometimes	Often	Always
• Dinner / Supper	Never	Rarely	Sometimes	Often	Always

The next 2 questions are about the food eaten in your household in the last 12 months and whether you were able to afford the food you need:

26. In the last 12 months, how often was this statement true for your household
"The food that we bought just didn't last, and we didn't have money to get more."

Often true	Sometimes true	Never true	Don't know

27. In the last 12 months, how often was this statement true for your household
"We worried whether our food would run out before we got money to buy more."

Often true	Sometimes true	Never true	Don't know

28. How much money do you spend each month on food for the members of your household?

Less than £150	£150 to £250	£250 to £700	£700 to £900	£900 or more

29. Do you intend to breastfeed, and If so for how long?

Not going to	*For up to 1 month*	*For up to 2 month*	*For up to 3 month*	*For up to 4 month*	*For up to 5 month*	*For up to 6 month*	*More than 6 month*	*Don't know*

30. Overall, how healthy do you think your <u>partner's</u> lifestyle is at the moment?

☹ *not at all* ☺ *Very healthy*☺

1	2	3	4	5	6	7	8	9	10

31. On average, how many times do you eat the following food each day?

(Please fill one number per line)		*Numbers of times a day*							
a.	**Fruit**	0	Rarely	1	2	3	4	5	6+
b.	**Raw vegetables** (e.g. lettuce ,tomatoes, salad)	0	Rarely	1	2	3	4	5	6+
c.	**Cooked vegetables**, not including potatoes (e.g. carrots, courgettes, broccoli)	0	Rarely	1	2	3	4	5	6+
d.	**Red meat** (e.g. beef, lamb and pork)	0	Rarely	1	2	3	4	5	6+
e.	**Processed meats** (e.g. hotdogs/burger/bacon/ham/sausage)	0	Rarely	1	2	3	4	5	6+
f.	**Wholemeal Cereals grains** (e.g. bread, buckwheat, rice, or pasta)	0	Rarely	1	2	3	4	5	6+
g.	**Legumes** (e.g. lentils, chick peas, soy, beans etc.)	0	Rarely	1	2	3	4	5	6+
h.	**Fish or fish products** (e.g. Cod, tuna, fish fingers)	0	Rarely	1	2	3	4	5	6+
i.	**High fat processed food** (e.g. cream, chips, fried food)	0	Rarely	1	2	3	4	5	6+
j.	**High sugar food** (e.g. sweets, cakes, cookies, chocolates)	0	Rarely	1	2	3	4	5	6+
k.	**Nuts**	0	Rarely	1	2	3	4	5	6+
l.	**Breakfast cereal** (any kind of processed cereal like cornflakes etc.)	0	Rarely	1	2	3	4	5	6+
m.	**Crisps or other savoury snacks**	0	Rarely	1	2	3	4	5	6+
n.	**Alcoholic drinks**	0	Rarely	1	2	3	4	5	6+
o.	**Coffee or black tea**	0	Rarely	1	2	3	4	5	6+
p.	**Low calorie or diet drinks**	0	Rarely	1	2	3	4	5	6+
q.	**Sweet beverages** (e.g. squash, fruit drinks, fizzy drinks)	0	Rarely	1	2	3	4	5	6+
r.	**A glass of water**	0	Rarely	1	2	3	4	5	6+

Details about yourself:

Please complete the following section about yourself.
Your responses will be kept strictly confidential and are important to help us to analyse the questionnaire.

1. **Gender:** Male / Female / Other / Prefer not to say**.**
2. **Date of birth:** ____________
3. **My religion or belief:** No religion / Christian / Hindu / Jewish / Muslim / Sikh / Other religion____
4. **Postcode:** _____________
5. **Marital status:** Married / Living with partner / Long term relationships / Single / Divorced / Other _______
6. **What is your height?** ________ cm
7. **What is your weight (**pre-pregnancy**)?** ________ Lb.
8. **How many weeks pregnant are you?** ______ Weeks.
9. **What is your monthly household income before tax and including benefits?**
   (for example - pensions, working family tax credit and/or jobseekers allowance etc)
   - ☐ Less than £500 a month.
   - ☐ Between £ 500 and £ 2000 a month.
   - ☐ Between £ 2,000 and £ 3,000 a month.
   - ☐ Between £ 3,000 and £ 4,000 a month.
   - ☐ Between £ 4,000 and £ 5,000 a month.
   - ☐ More than £5,000 a month.
   - ☐ Prefer not to say.
10. **Medical conditions-you may have:**
    - ☐ No Medical conditions.
    - ☐ Diabetes.
    - ☐ Obesity.
    - ☐ High blood pressure.
    - ☐ Other ____.
11. **How satisfied are you with your health?**
    1. Very dissatisfied / 2.Dissatisfied /3. Neither satisfied nor dissatisfied / 4.Satisfied / 5.Very satisfied
12. **What is the highest level of education you have completed?**
    No formal schooling / Less than primary school / Primary school completed / Secondary school completed / High school completed / College/University completed / Post graduate degree / other: _____

We would be very glad to know anything else you would like to tell us about yourself or your family's nutrition:

____

____

____

____

____

* * *

Thank you for taking the time to complete this questionnaire !
